# Factors Driving China’s Carbon Emissions after the COVID-19 Outbreak

**DOI:** 10.1021/acs.est.3c03802

**Published:** 2023-11-16

**Authors:** Xinlu Sun, Zhifu Mi

**Affiliations:** † The Bartlett School of Sustainable Construction, University College London, London WC1E 7HB, U.K.

**Keywords:** CO_2_ emissions, input−output analysis, structural decomposition analysis, pandemic impacts, green recovery

## Abstract

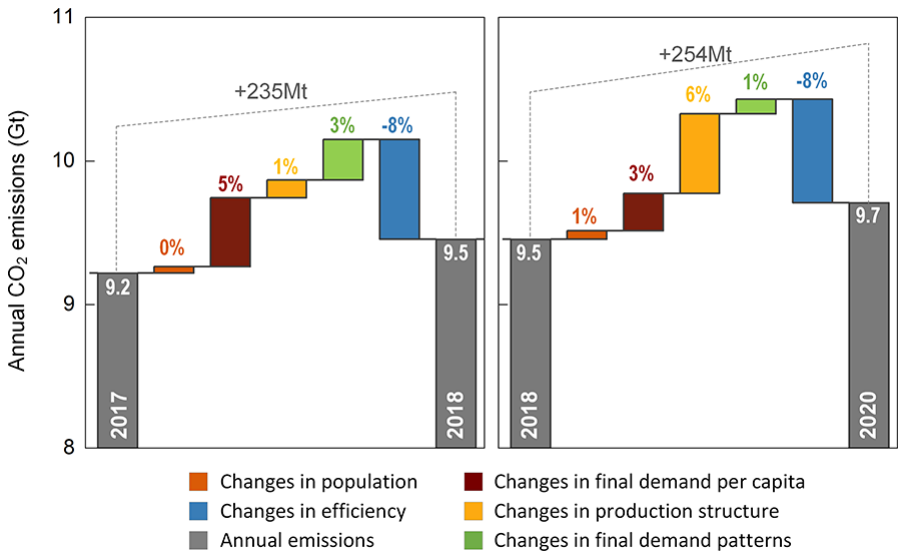

The outbreak of the coronavirus disease 2019 (COVID-19) may exert profound impacts on China’s carbon emissions via structural changes. Due to a lack of data, previous studies have focused on quantifying the changes in carbon emissions but have failed to identify structural changes in the determinants of carbon emissions. Here, we use China’s latest input–output table and apply structural decomposition analyses to understand the dynamic changes in the determinants of carbon emissions from 2012 to 2020, specifically the impact of COVID-19 on carbon emissions. We find that final demand per capita contributed to emissions growth at a slower pace, but production structure drove a greater carbon emissions increase than before the pandemic. Export-led emissions growth rebounded, and investment-led emissions were more concentrated in the construction sector. The carbon intensity of several heavy industries increased, e.g., the nonmetallic products sector, the metal products sector, and the petroleum, coking, and nuclear fuel sector. In addition, lower production efficiency and increased reliance on carbon-intensive inputs indicated a deterioration in production structure. For policy implications, efforts should be undertaken to increase investment in low-carbon industries and increase the proportion of consumption in GDP to shift investment-led growth to consumption-led growth for an inclusive and green recovery from the pandemic.

## Abstract 2

This study reveals energy efficiency loss in heavy industries and reliance on carbon-intensive inputs in China after the COVID-19 outbreak.

## Introduction

The COVID-19 pandemic swept the globe and exerted a profound impact on the global economy by halting economic activities in most countries. In response to the pandemic, China imposed drastic measures, including locking down most of its cities for more than two months in the first quarter (Q1) of 2020. This led to a shrinkage of the economy by 6.8% in 2020 Q1, which was the first contraction since 1992.^1^ By the summer of 2020, the halted economy was gradually reopened because widespread community transmission was eliminated in China, and travel restrictions were largely eased. Consequently, China rebounded from the contraction in the first half of the year and its economy expanded by 2.3%, becoming the only major economy to grow in the pandemic-ravaged year.

The changes in economic activities also caused a steep drop and then a strong rebound in carbon emissions. Many studies have found that COVID-19 greatly curtailed carbon emissions in the first half of 2020 in China. These studies focused on quantifying the emission changes at the sectoral or national level. Han et al.^2^ found that lower coal consumption in secondary industry and cement production led to declines in carbon emissions in 2020 Q1. Norouzi et al.^3^ found effects on electricity and petroleum demand, which may be magnified through the global supply chain.^4^ However, the short-term impact of the pandemic and declining carbon emissions was offset once the economic recovery began. Zheng et al.^5^ revealed that China’s CO_2_ emissions fell by 11.5% between January and April 2020 compared to the same period in 2019 and then rebounded to pre-pandemic levels due to the fast recovery of economic activities. Curtailed carbon emissions via halted economic activities and the collapse in demand were therefore temporary, and a rebound has been witnessed with the easing of lockdown policies. However, the possible structural changes of carbon emissions that may exert profound impacts and drive long-term transitions urgently need to be identified.^6,7^

Changes in consumption patterns, energy preferences, production structure, and investment policies may have already altered the patterns of the driving factors of the carbon emissions. In some respects, positive effects have been witnessed, including changes in consumption behavior toward less carbon-intensive sectors.^8^ For example, lockdown policies have reshaped consumption patterns and boosted the development of the internet and online shopping industries, while energy consumption in traditional manufacturing and transport sectors has greatly decreased.^9^ In addition, the demand for renewable energy has accelerated, but fossil fuel has become less preferred.^2,10^ The power mix shifted toward renewable energy. The lockdown measurements led to a large reduction of coal-fired power generation, and renewables maintained a high share even with the release of the confinement.^11^ In other respects, the negative impact could offset previous carbon abatement efforts. Conceivably, the willingness of governments and companies to reduce carbon emissions could be largely diminished by the pandemic in light of the urgency to achieve robust economic recovery.^12^ Therefore, investment may be targeted in carbon-intensive infrastructure. Falling energy demand retards the growth of renewable energy installation. This could be compounded by the collapse in oil prices, which increases the allure of fossil fuels in economic recovery. The impacts of the changes in production structure remain to be quantified. On the one hand, production structures were altered because of the increased demand in pharmacy industries and the drop in the economic activities of services, construction, and some manufacturing sectors in early 2020.^13^ On the other hand, the rebound in China’s carbon emissions in 2020 was initially driven by coal power, cement, and other heavy industries.^14^ These factors acting in utterly different directions could have structural impacts and change the determinants of carbon emissions.

It is of interest to systematically investigate the structural changes in carbon emissions in China for timely and targeted policy interventions. The structural changes due to COVID-19 have larger impacts on the environment than on macroeconomics.^13^ The urgent detection of such changes could assist in identifying and modifying policies that are less effective in achieving green recovery and derive policy implications to avoid carbon-intensive development trajectories.^7^ Currently, companies are suffering a multitude of challenges, such as a deterioration in demand, interruptions in the supply chain, revocation of export orders, a shortage of raw materials, and distortion in transportation networks.^15^ Wang et al.^16^ warned of the risk of deterioration in energy efficiency when recovering from the hardship. There is growing consensus that the socioeconomic impact of the COVID-19 pandemic is far more severe than that of the 2008 financial crisis.^10,12,17^ The financial crisis made profound changes to China’s economic transition process and carbon emissions by decreasing the contribution of exports to the GDP^18^ and increasing carbon emissions because of the carbon-intensive economic stimulus strategy.^16,19,20^ Compared with the financial crisis, the economic crisis associated with the pandemic is more deeply connected with individual behavior. The impact of COVID-19 is also different, with unprecedented speed and severity.^21^ Therefore, the structural impact of COVID-19 should be identified as early as possible to identify inappropriate recovery patterns and to implement targeted adjustment and interventions to prevent structural deterioration.

However, previous studies have failed to systematically explore the structural changes in carbon emissions due to the lack of data. Recent studies tried to quantify the emission changes after the COVID-19 outbreak,^22^ investigate sectoral emission trends, such as carbon emissions from aviation^23^ and municipal solid waste treatment,^24^ or analyze the impact of COVID-19 on China’s carbon emissions from perspectives of distributions among provinces.^25^ These studies assist in improving temporal or spatial resolution or providing insights into sector-specific emission changes. But the direct and indirect relationships between sectors and industries need be revealed with input–output (IO) tables. An IO table can describe the sector-by-sector transformation process. Based on the input–output analysis, structural decomposition analysis (SDA) has been widely used to track changes in energy consumption or carbon emissions.^26^ By decomposing the changes of carbon emissions into the product of changes in several driving factors, SDA is advantaged to quantify the contribution of each factor to the changes in carbon emissions. Because of the great structural details in the IO table, carbon emissions induced both from direct demand of a sector and those impacted by the supply chain can be analyzed. SDA can distinguish between several technological and structural effects and analyze socio-economic drivers from both production and consumption perspectives.^27^ Therefore, SDA is effective for systematically analyzing the changes in the driving factors of carbon emissions after the COVID-19 pandemic, but the time lag of IO data caused difficulty in conducting such analyses until the latest IO table in 2020 was launched.

In this study, we used the latest-released IO table of China in 2020 and applied the SDA method to understand the dynamic evolution of the driving forces of China’s carbon emissions from 2012 to 2020. In particular, we analyzed the structural changes in carbon emissions from 2018 to 2020 to investigate the impact of the COVID-19 pandemic. With the latest IO table of China’s economy, we are able to reveal the structural impact of COVID-19 and to identify the changes in the determinants of China’s carbon emissions. The dynamic changes in five socioeconomic factors that drive changes in the increase of carbon emissions, including population, energy efficiency, production structure, final demand patterns, and final demand per capita, are analyzed in the period under consideration. The results could reveal the structural changes after the outbreak of COVID-19 by considering the relationships between sectors. The emission changes caused by changes in production efficiency, energy mix, consumption and investment patterns, and exports are analyzed. This study can quantify the contribution of different driving factors to changes in carbon emissions and therefore assist in timely and effective policy adjustments to prevent structural deterioration.

## Methods

### Environmental Input–Output Analysis and Structural Decomposition Analysis

Input–output analyses were originally developed by Wassily Leontief in the 1930s to delineate the economic linkage among industries by quantifying the input and output flow.^28^ The framework was expanded to a broader field by simply adding a column to describe the resource or emission intensity of each sector, including carbon emissions, energy consumption, and other environmental topics.^29^ This is known as the environmental input–output analysis (EIOA). The fundamental theory of the EIOA is shown in eqs 1 and 2:
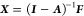
1where ***X*** = (*x*_*i*_) is the vector of the total output and *x*_*i*_ is the total output of sector *i*, ***I*** is the identical matrix, and (***I****–****A***)^*–*^^1^ is the Leontief inverse matrix. The matrix ***A*** = (*a*_*ij*_) is the technical coefficient matrix, and *a*_*ij*_*= z*_*ij*_/*x*_*j*_, in which *z*_*i*__*j*_ is the monetary input of sector *j* from sector *i*. In the final demand matrix, ***F*** = (*f*_*i*_), *f*_*i*_ is the final demand for the products of sector *i*. In the IO table, the final demand is divided into five categories: urban household consumption, rural household consumption, government, fixed capital formation, and changes in inventories. The total output of a sector needs to fulfill the final demand and inputs demanded by all the sectors.
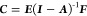
2where ***C*** is the matrix of total carbon emissions embedded in the goods and services used for the final demand and ***E*** is the vector of carbon emission intensity of all sectors, which is measured by carbon emissions per unit of economic output. Emissions induced by fossil fuel combustion and cement production are included in this study. Equation 2 shows the calculation of carbon emissions induced by the final demand, including rural and urban households, government, capital and changes in inventory stock, as well as exports.

SDA combines input–output analysis and decomposition analysis. SDA can quantitatively measure the contribution of each socioeconomic factor in driving the changes in both direct and indirect carbon emissions. The input and output linkages between the different sectors can be accounted for when identifying the direct and indirect impact of each driving factor. Therefore, SDA has been widely used to interpret the dynamic effects of socioeconomic drivers in the process of carbon emission abatement in different regions. Previous studies have explored the impact of socioeconomic drivers on China’s production-based carbon emissions as well as consumption-based emissions.^19,20,30^

The changes in national carbon emissions can be decomposed by SDA as follows:^19^
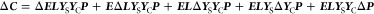
3where **Δ** denotes the change in a factor, ***L*** is the Leontief inverse matrix, ***L*** = (***I****–****A***)^*–1*^, ***P*** is the population, ***Y***_S_ is a column vector of the final demand patterns, and ***Y***_C_ is the final demand per capita. SDA can quantify the contribution of the changing factor to emission changes while all the other factors are held constant. As there are five factors, 5! = 120 equivalent decomposition forms can be obtained. Various methods have been proposed to execute the decomposition, including polar decomposition and midpoint weight decomposition.^26^ Given the pros and cons of different methods to address this issue, we take the average of all possible first-order decompositions and calculate the weights accordingly. A detailed discussion of this issue can be found in previous studies.^31,32^

We apply SDA to understand the changes in the driving forces of China’s carbon emissions from 2012 to 2020 at the national level. Here, we used China’s IO tables in 2012, 2017, 2018, and 2020. The five socioeconomic factors include population, final demand per capita, final demand pattern, production structure, and energy efficiency.

**Figure 1:**
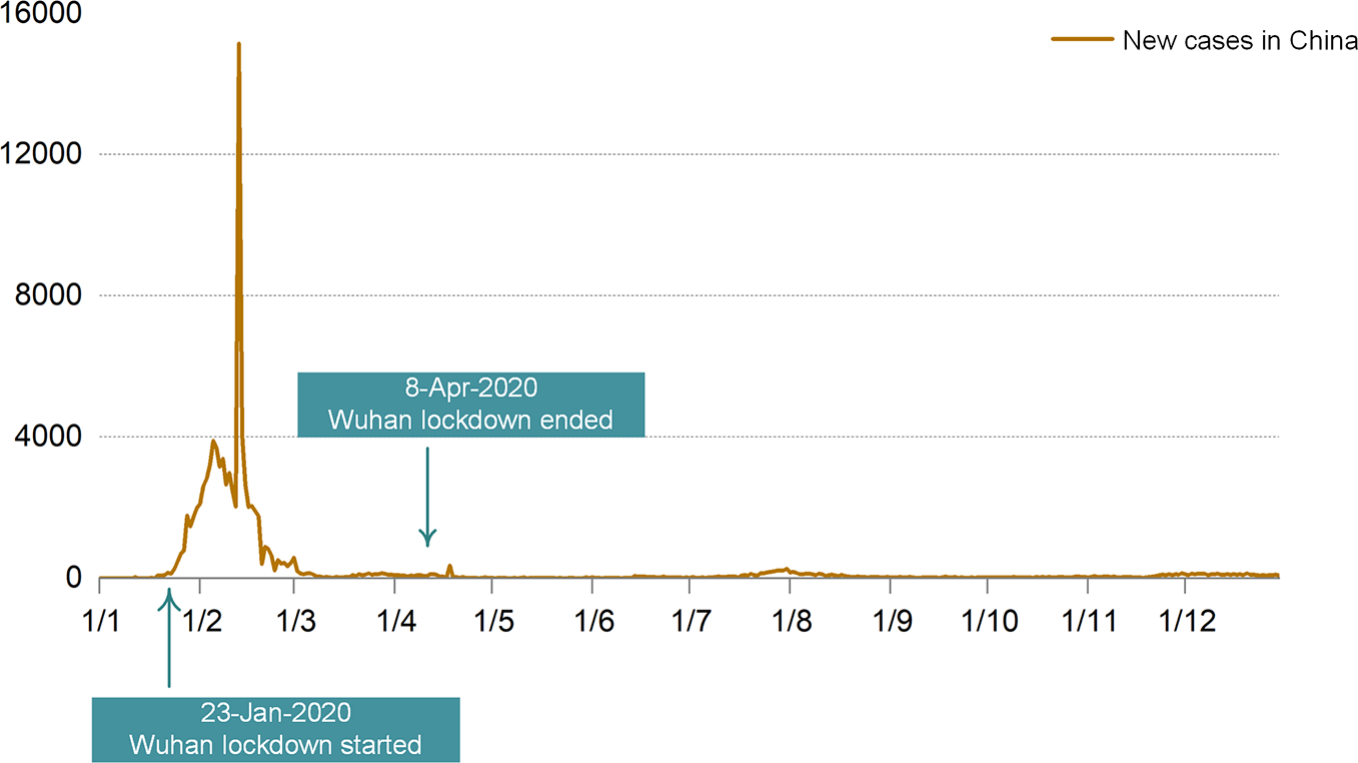
Daily new confirmed COVID-19 cases and lockdown in 2020. Source: World Health Organization, COVID-19 Dashboard (https://covid19.who.int/region/wpro/country/cn).

We divide the eight years into three stages according to the characteristics of carbon emission changes. The first stage is the entrance of the new normal phase, when carbon emissions plateaued (2012–2017). The second stage is the rebound stage before the pandemic, when carbon emissions started to increase again but at a low speed (2017–2018). The last stage is set to investigate the impact of the COVID-19 pandemic on the determinants of carbon emission changes in China (2018–2020). We compare the driving factors of carbon emissions before and after 2018 to indicate the structural changes that happened after the outbreak of COVID-19 (Figure 1 for the start and end of lockdown in 2020).

### Carbon Emission Inventories

We apply the administrative territorial scopes defined by the Intergovernmental Panel on Climate Change (IPCC) to develop China’s carbon emission inventories.^33,34^ The administrative territorial emissions, also known as production-based emissions, indicate emissions taking place within the territories of a particular jurisdiction.^33,34^ Carbon emissions from both fossil fuel consumption and cement production are calculated in this study. Emissions from international aviation or shipping are not accounted for. Sectoral emissions induced by fossil fuel combustion, ***C***_e_, are calculated as
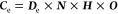
4where ***D***_e_ denotes unit fossil fuel consumption, with missing or double accounting avoided. ***N × H × O*** is the emission factor for fuel combustion calculated by three product terms, with the net calorific value measuring heat released from unit fossil fuel represented by ***N***, the carbon content representing CO_2_ emitted from unit released heat represented by ***H***, and the oxygenation calculating oxidization rate of fossil fuel combustion represented by ***O***. Sectoral energy consumption of 26 types of fossil fuel types are collected from National Energy Statistical Yearbook. The 26 fuel types are merged into 17 types according to the literature.^35,36^ Fossil fuels used for nonenergy use and energy loss during transportation were removed from total energy consumption, as follows:
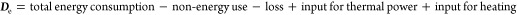
5

Carbon emissions released during the industrial process in cement production, ***C***_p_, are calculated as
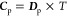
6where ***D***_p_ denotes the amount of cement production and *T* is the emission factor for the cement process, measured by CO_2_ emitted in unit cement production as 0.2906 ton of CO_2_ per ton of cement.^37^

### Linking Imports to the Global Multiregional Input–Output Model

In this study, carbon emissions embodied in China’s imports are calculated by linking to the global multiregional input–output (MRIO) model. One possible approach is to adopt the carbon intensity of China’s production sector. However, this accepts the assumption that the technologies used to produce China’s imported goods and services are at the same level as China’s domestic production. This causes large errors because the carbon intensity in China is usually higher than the global average. Therefore, we link China’s imports to the global multiregional input–output model. The widely used EXIOBASE database is used here, and China’s imports in each sector and by each final demand agency are divided into all other regions according to the EXIOBASE MRIO tables in the corresponding year. We coordinate the sectors in China’s IO tables and the global MRIO tables. Finally, the linked MRIO model includes the economic flows of 20 sectors in China and 48 other regions in the world. The carbon emissions embodied in imports are calculated as follows:
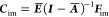
7where ***C***_i__m_ represents the embodied carbon emissions in imports; ***E*** is a row vector of carbon intensities for all sectors in all regions; ***A*** is the direct requirement matrix among all sectors in all regions; and ***F***_i__m_ is a column vector of China’s imports from all sectors in all regions, including the consumption of both intermediate inputs and final demands.

### Data Sources

The data sets used in this paper are all publicly accessible and easily downloadable through database websites. China’s IO tables and population data are published by the National Bureau of Statistics of China, and the energy consumption data are derived from the National Statistics Yearbook.^38^ The global MRIO tables are obtained from the EXIOBASE database.^39^ All IO tables are deflated to 2020 constant prices. The exchange rates of Euro and RMB are from the World Bank database.^40^ Carbon emission inventories are not published officially. We therefore use the national energy balance sheet, energy consumption data of each industry, and cement production data derived from the website of the National Energy Statistics Yearbook, National Statistics Yearbook and China Emission Accounts and Datasets (CEADs) (www.ceads.net) to establish China’s emission inventories. The emission factors and the concordance of the sectors in the MRIO tables, the energy consumption data sets, and the 20 sectors in the IO tables used in the analysis are derived from previous studies (Tables S1 and S2).^19,20^

### Uncertainties and Limitations

In this study, we focus on the early stage impact of COVID-19 at the national level as the latest IO table used in this research is from 2020. This leads to uncertainties and limitations in this study. First, the long-term and lag effects of COVID-19 on carbon emissions are difficult to explore in this study. As the latest IO table is in 2020 currently, the revealed structural changes in this study are the early stage impacts of the COVID-19 outbreak. Studies in the future using data in the later years could reveal the potential long-lasting behavioral and social changes as a result of the pandemic. Second, limitations in the precision of data may cause uncertainty. Trends in carbon emissions are analyzed based on annual data, as is presented in the Slowed Growth of Carbon Emissions section, while the impact of the outbreak of COVID-19 on the driving factors of emission changes is analyzed based on comparison between the last period, 2018–2020, and previous periods. It would be more appropriate to use data in 2019–2020 to reveal the impact of COVID-19, but the IO table in 2019 is inaccessible. In addition, high-resolution analysis, e.g., analysis based on monthly or daily data, would also complement this study when data is available. Third, as subnational institutions are showing increasing significance in combating climate change, provincial- and city-level studies can reveal insights into subnational decarbonization issues. This can be improved in the future when data is available. Fourth, uncertainties also come from the assumptions of the EIOA method and the SDA method. Inherent uncertainties from the EIOA method include assumptions of linear relationships between the sectors and homogeneous products in each sector. Regarding the SDA method, information loss caused by sector and temporal aggregation and the decomposition method bring uncertainties in the results.^27^ We conduct an uncertainty analysis to show the robustness of the results (see the uncertainty analysis in Figures S1 and S2).

## Results

### Slowed Growth of Carbon Emissions

The growth of carbon emissions was further slowed down after the COVID-19 outbreak. In 2020, the total carbon emissions in China were 10.1 Gt, increased by 0.8% compared to the 2019 level. The growth rate was lower than the pre-pandemic level, as carbon emissions increased by 1.7%, 2.4%, and 1.6% in 2019, 2018, and 2017, respectively. Studies show that carbon emissions dropped dramatically in the first half of 2020 in China due to lockdown measurements and halted economic activities,^5,7^ but the emissions rebounded robustly with recovery from the pandemic.

**Figure 2:**
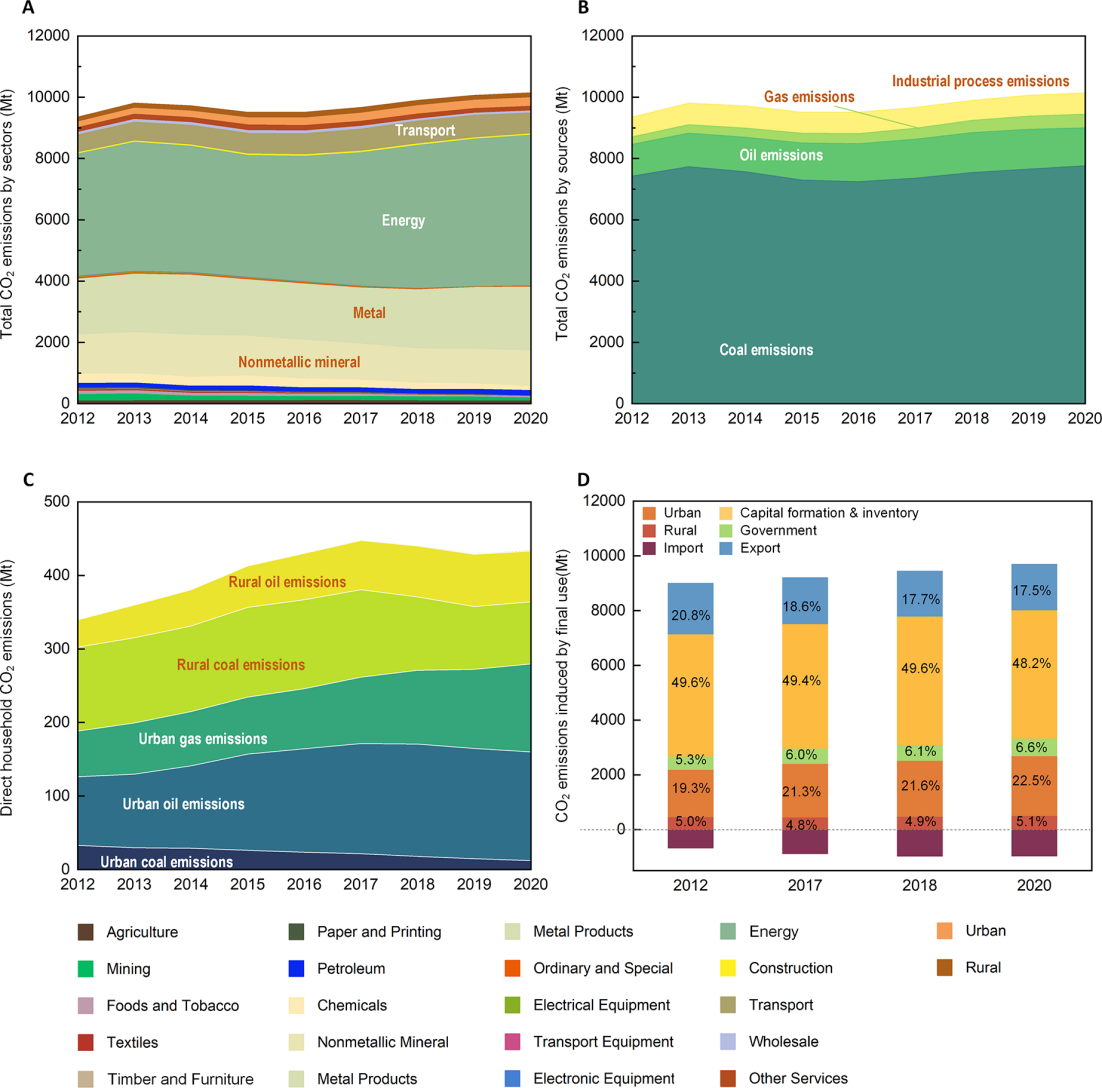
Trends of China’s carbon emissions from 2012 to 2020. (A) Trends of carbon emissions by sectors. (B) Trends of carbon emissions by fuel. (C) Direct household CO_2_ emissions in China. (D) CO_2_ emissions induced by different final users (rural consumption, urban consumption, government consumption, capital formation, inventory changes, and exports).

The energy sector (electricity, gas, and water) was the major source of carbon emissions, the emissions of which increased by 2.3% compared to that in 2019 and accounted for 48.5% of total national carbon emissions in 2020 (Figure 2). The proportion of the carbon emissions of the energy sector continued growing from 41.9% in 2016 to almost half in 2020, indicating the urgency to decarbonize energy production, especially power generation. Power demand grew at a lower rate in 2020 compared to the pre-pandemic level, with a slowed increase in renewable energy (Figure S3). In 2020, the total consumption of electricity was 7.8 trillion kWh, increased by 3.7% from the 2019 level. Before the pandemic, power consumption had grown at an annual rate of 6.0% since the new normal. As for the energy mix, renewable energy grew by 11.1% annually during 2012 to 2019, while in 2020, the growth rate was only 7.3%. This indicates a slow down in renewable energy growth, which is also found in the literature.^41^ The proportion of carbon emissions in most manufacturing sectors declined, consistent with the pre-pandemic trend. For example, the carbon emissions of the chemicals sector accounted for 1.3% of the total emissions in 2020, decreased from 1.8% in 2019. On the other hand, the proportion of carbon emissions in some carbon-intensive sectors has slightly increased or remained unchanged; for example, the petroleum, nonmetallic mineral products, and metal products sectors emitted 1.8%, 11.5%, and 20.5% of the total emissions in 2020, respectively.

Coal remained the largest source of carbon emissions in China (Figure 2B), driven by growing electricity demand. In 2020, 52.3% of total coal consumption was used for thermal power generation, which is the same proportion as the previous year. Decoupling economic growth from coal combustion and fostering renewable energy development is one of the most challenging tasks in China’s carbon reduction. Emissions from oil combustion decreased by 5% in 2020 because of a sharp drop in travel demand. Oil-related carbon emissions were 1236.6 Mt and accounted for 12.2% of the total emissions. This can be seen from the shrunk usage of gasoline in the transport sector and the private sector (urban and rural households). After continuous growth, gasoline demand declined dramatically by 10.8% and 3.2% in the transport sector and the private sector in 2020.

The downward trend of household direct emissions was reversed after the COVID-19 outbreak (Figure 2C). A slight increase in household carbon emissions from direct usage of fossil fuels occurred in 2020, after a two-year decline from 2017 to 2019. The previous decline was mainly a result of reduced coal usage in rural areas. Rapid urbanization in China has led to the migration of a large number of rural populations to urban areas, contributing to reduced rural carbon emissions. In addition, a clear energy transition from coal to electricity in rural areas can be seen before the pandemic (see the Supporting Information). From 2017 to 2019, rural coal-related emissions declined by 28.1%, while gas-related emissions grew by 83.6%. However, COVID-19 slowed the energy transition in rural areas, evidenced by a slight decrease of 1.1% in rural coal-related emissions and an increase of 28.0% in gas-related emissions. Changes in urban coal- and gas-related emissions followed the trend before the pandemic. Despite the drop in travel demands and related oil consumption, heightened levels of homebound activity due to travel restrictions led to increased demand in residential heating and cooking. Therefore, urban and rural household demand in liquefied petroleum gas (LPG) usage increased slightly to 28.6 Mt in 2020 after a two-year decline.

### Efforts Required to Improve Energy Efficiency of the Heavy Industries

The impact of the COVID-19 outbreak on carbon emissions is interpreted by comparing the driving factors before and after 2018. The main findings include decelerated energy efficiency improvement, increased export-induced emissions, and a rebound in carbon-intensive production. The three main findings are analyzed in this subsection and the following two subsections.

**Figure 3:**
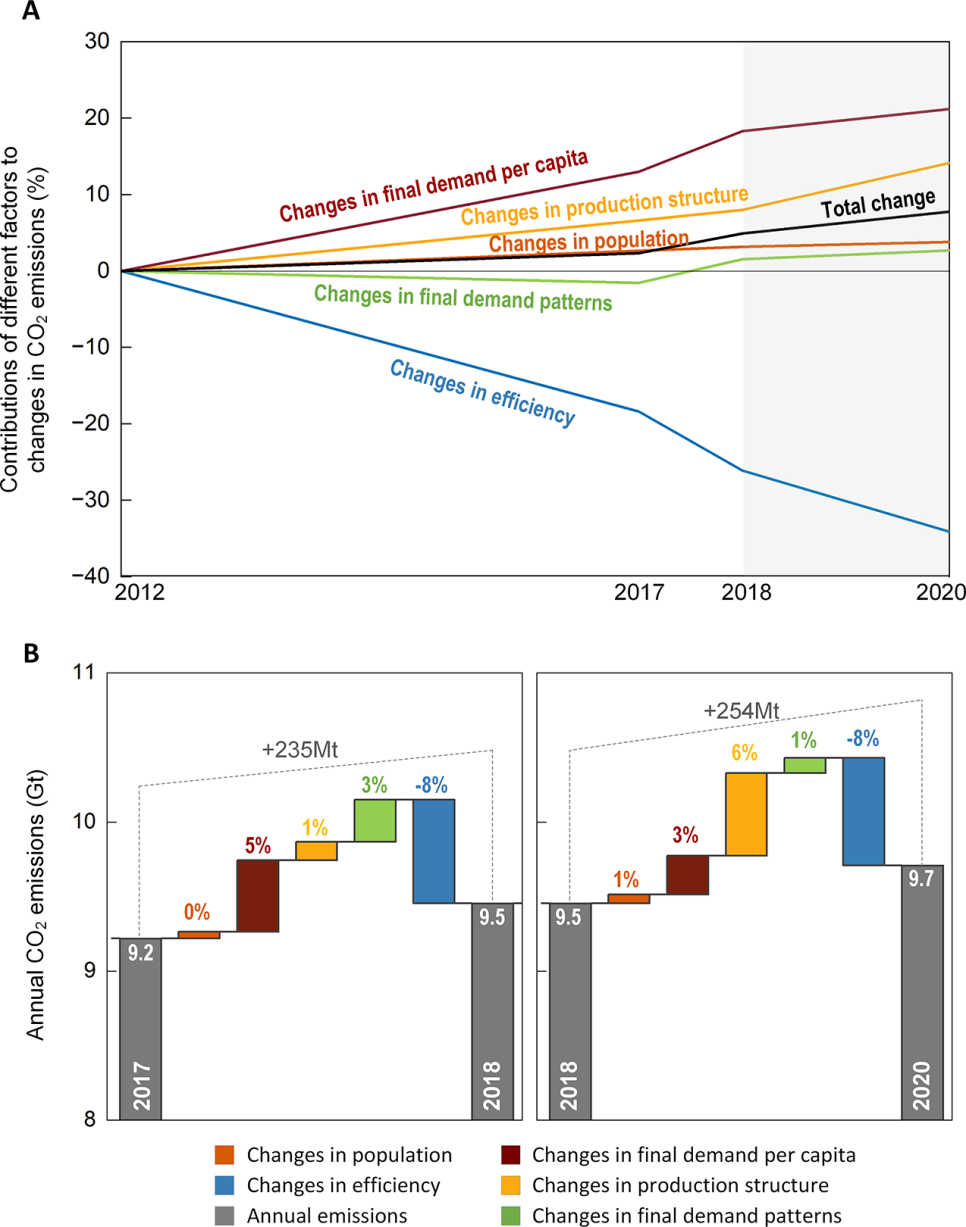
Trends of the drivers of carbon emissions from 2012 to 2020. (A) Contributions of different factors to changes in Chinese CO_2_ emissions between 2012 and 2020, taking 2012 as the base year. (B) Absolute contributions of different factors to changes in Chinese CO_2_ emissions for 2017–2018 and 2018–2020.

The contribution of energy efficiency to China’s decarbonization from 2018 to 2020 remained at the same level in 2012–2017, reducing carbon emissions by 7.6% (Figure 3). Although there is a slight decrease compared to the contribution during 2017 to 2018, it remained relatively stable during the whole period from 2012 to 2020. In 2017–2018, the improvement of energy efficiency accelerated, with a contribution rate to carbon reduction of 7.5%. In this year, a hastened decline in the carbon intensity of many sectors can be observed. For example, the carbon intensity of the energy sector decreased by 8.4% in 2018. Efficiency gains were even greater in some manufacturing sectors. Carbon intensity declined by more than a third in the timber and furniture sector (51.7%), the transport equipment production sector (46.8%), and the textiles sector (37.0%). Nonetheless, the energy efficiency improvement was decelerated after the COVID-19 outbreak, and the annual contribution rate of efficiency gains to carbon reduction dropped to 3.9% in 2018–2020 because decarbonization of most sectors slowed again in the pandemic era. The long-term trend in the driving factors from 2002 is provided in the Supporting Discussion.

**Figure 4:**
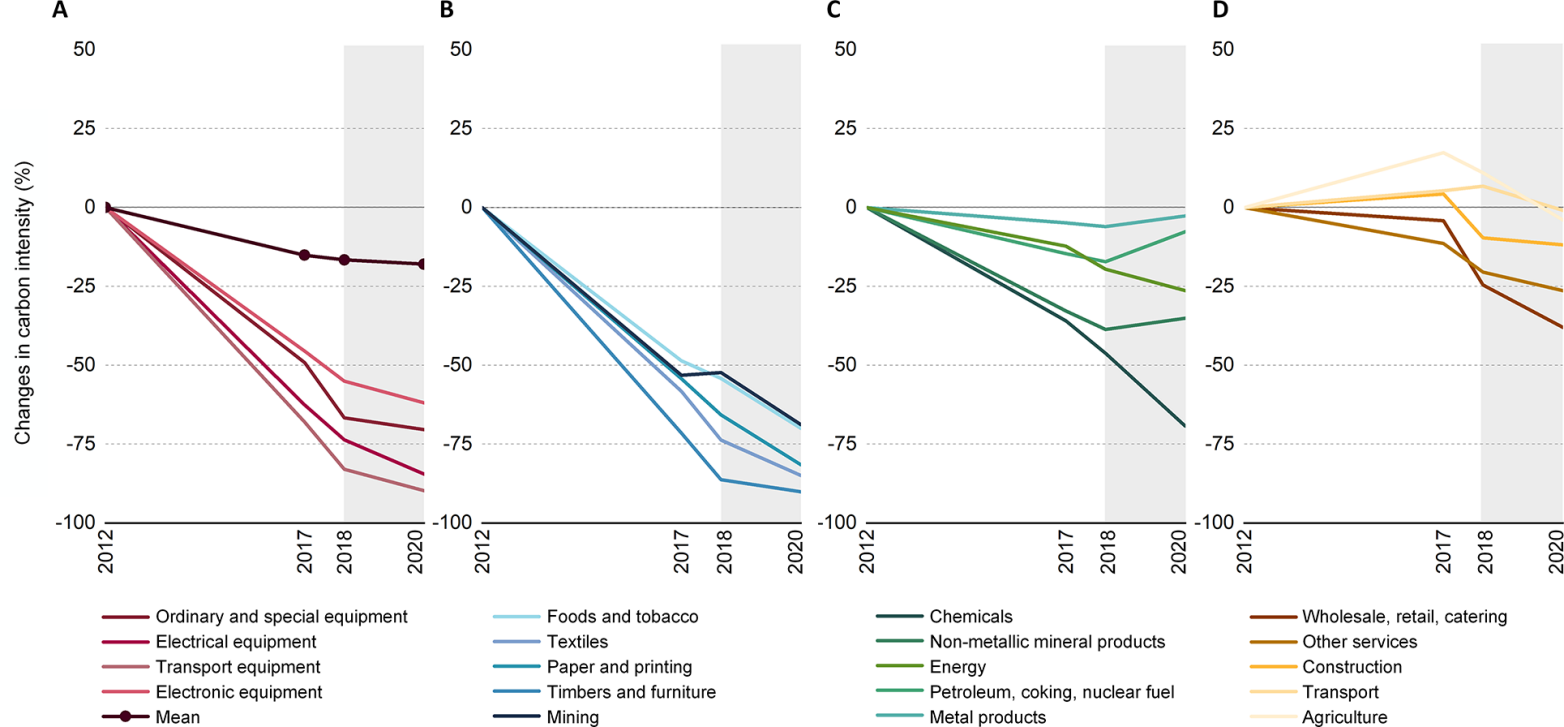
Changes in carbon intensity for all sectors from 2012–2020 in China. (A) Trends in carbon intensity for the nation and for the equipment manufacturing sectors. (B) Trends in carbon intensity for the mining and some manufacturing sectors. (C) Trends in carbon intensity for the energy and heavy-industry-related sectors. (D) Trends in carbon intensity for the tertiary industry, agriculture, and construction sectors.

The mining sector, the chemical sector, and some manufacturing industries maintained rapid decarbonization. To be specific, the carbon intensity of the mining sector, the chemical sector, the paper and print sector, and the electrical equipment sector declined by 34.7%, 42.9%, 46.1%, and 41.1% from 2018 to 2020, respectively. The carbon intensity of some other manufacturing sectors also continued to decline, although the pace of decline had slowed down. The ordinary and special equipment sector shows a rapid decarbonization with an annual carbon intensity reduction of 8.9% before the pandemic, while the intensity reduction rate was 5.8% annually in 2020. Despite the continuous improvement in energy efficiency, the total carbon emissions of the mining and chemical sectors and all the manufacturing sectors (Figures 4A and B) only accounted for 3.2% of China’s total carbon emissions in 2020.

The improvement of energy efficiency in the heavy and carbon-intensive industries is especially vital but encountered obstacles after the COVID-19 outbreak (Figure 4). The carbon intensity of several sectors even increased in 2020 after continuous decline since 2012. For instance, the carbon intensity of the petroleum, coking, and nuclear fuel sector, the nonmetallic mineral products sector, and the metal products sector declined by a total of 7.4%, 73.1%, and 37.1% from 2012 to 2018, respectively, but increased by 11.5%, 5.8%, and 3.6% from 2018 to 2020, respectively. In addition, the energy sector is also among the most carbon-intensive sectors. The carbon intensity reduction of the energy sector slowed down to an annual rate of 4.3% after the COVID-19 outbreak compared to an 8.4% decrease in 2018. These four sectors accounted for 82.3% of total carbon emissions, indicating the necessity to improve the energy efficiency of these key sectors. Achieving the decoupling of economic growth from carbon emissions requires the decarbonization of key sectors. However, due to plateaued progress in energy efficiency improvements in the carbon-intensive sectors, the carbon intensity per unit of GDP decreased by 1.4% in 2020.^42^ Compared to a 4.4% decline in 2019, this indicates a slower pace of decoupling economic growth from carbon emissions than the pre-pandemic level.

### Increase in Export-Induced Emissions

The COVID-19 pandemic exerts a direct impact on China’s carbon emissions by weakening final demand, i.e., GDP growth. The annual contribution of final demand per capita to the carbon emission increments was sharply reduced to 1.4% from 2018 to 2020, much less than the average level in the new normal (2.8%). The contribution of the final demand pattern to emission growth in 2018–2020 (1.1%) was lower than that in 2017–2018 (3.05%). Overall, household consumption was yet to recover from the recession because of the pandemic while economic growth in 2020 was supported by investment and export. Household consumption and export increased by 4.4% and 7.1%, respectively, from 2018 to 2020, indicating unbalanced expansion in private consumption and export. It is also revealed that investment and export contributed to a 1.8% and 0.6% increase in GDP in 2020, respectively, while private and public consumption decreased GDP by 0.2%.^42^ Population has steadily driven the growth of carbon emissions in China for decades, but the contribution has become smaller in recent years. In 2018–2020, the rise in population drove 0.6% growth in carbon emissions, and in 2012–2017, the contribution was 2.7%. With the peak of population in 2022, the contribution may be reversed in the coming years.

**Figure 5:**
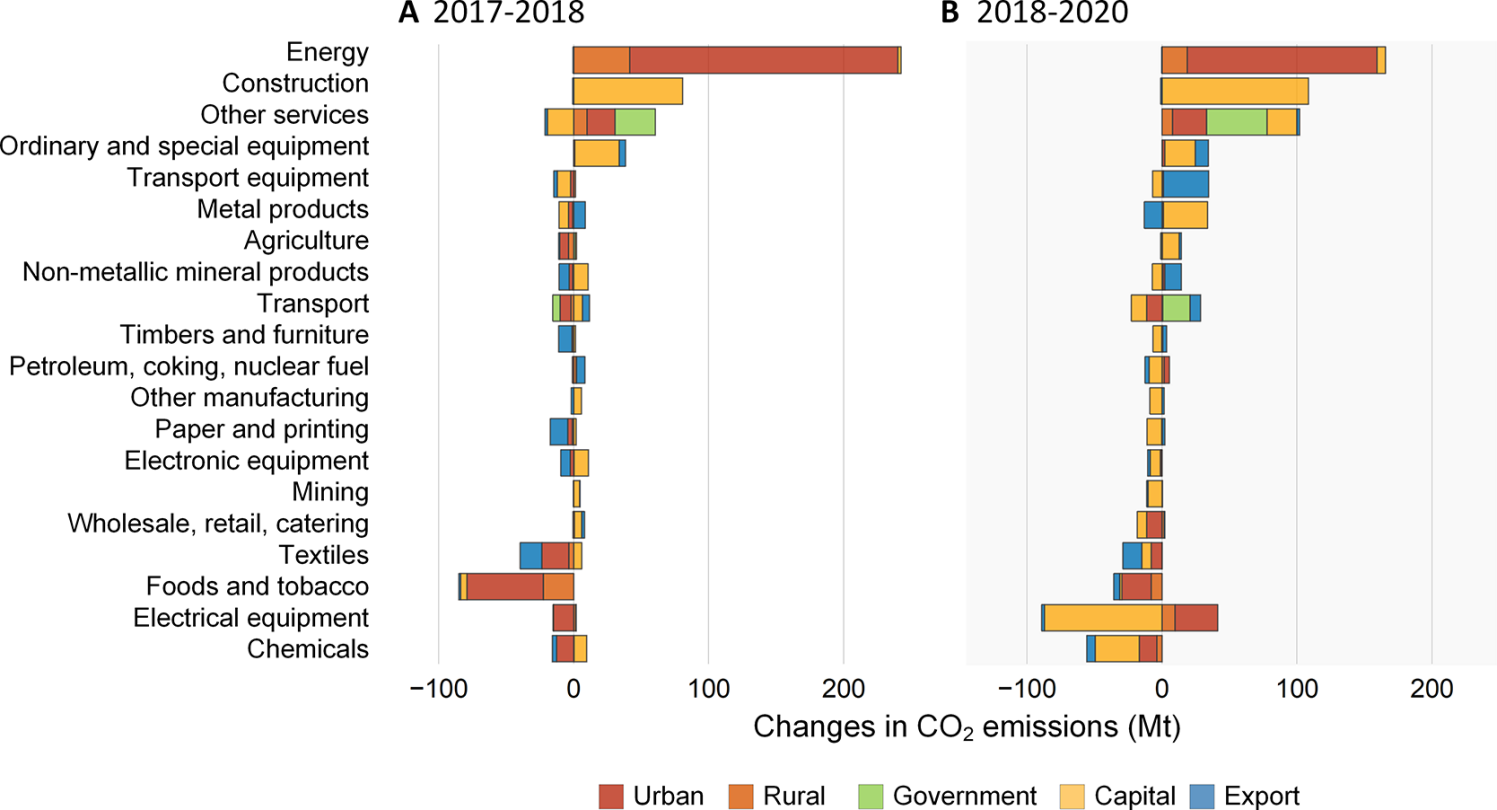
Contributions of different sectors and final uses to Chinese CO_2_ emissions growth. A and B show the results for 2017–2018 and 2018–2020, respectively.

A rebound in export-supported emission growth and a trend of more concentrated sector-based investment was shown after the COVID-19 outbreak (Figure 5). Overall, carbon emissions embodied in exports peaked and plateaued since 2012 in China, despite continuous growth in export value, while emissions embodied in imports had increased significantly before the outbreak of COVID-19. The spread of the pandemic worldwide in 2020 changed the trade situation. Export-induced carbon emissions rebounded by 1.7% from 2018 to 2020 (Figure S4). The dominant contribution to the growth of export-induced emissions was from the export of transport equipment (33.4 Mt). Exports of nonmetallic mineral products and ordinary and special equipment also led to increases in carbon emissions (12.4 and 9.7 Mt, respectively). On the contrary, import value and emissions embodied in imports both decreased. From 2018 to 2020, import shrunk by 1.4% while import-induced emissions slightly dropped by 0.8%. The changes in export and import were highly related to COVID-19 cases.^43^ Overall, the effective COVID-19 containment endowed China with international trade resilience.^44^ The changes in export and the embodied emissions can be explained both from production and consumption perspectives. From the production side, the well-controlled cases in China led to a robust recovery of China’s economic activities in the second half of 2020. The halted industrial production in the first quarter gradually rebounded after the second quarter as lockdowns eased, and this enabled China to recover its production in response to the demand for exports. From the consumption side, the pandemic situations in its trade partners boosted export demands in China.^45^ The shrink in imports and related emissions could be explained to some extent by weak domestic demand, the insufficient supply from other countries, and the price deflation of energy and other bulk commodities.^46^

The stimulus package for economic recovery from the pandemic led to more concentrated investment in specific sectors, especially the construction sector. In 2020, the Chinese government released a series of fiscal and monetary policies to stimulate the contracted economy, targeting tax breaks, consumer subsidies, and infrastructure investment. The new infrastructure construction plan has become a strategy to achieve the goals of both stimulating job creation and reviving a flagging economy. Investment in key segments has been accelerated, including industrial internet, 5G networks, smart city, intelligent transportation, and artificial intelligence. These stimulus measures helped China escape the economic recession but also led to a rebound of carbon emissions in the construction sector. The proportion of investment in the construction sector to the total investment increased from 63.9% to 65.4% in this period. Consequently, the investment-induced carbon emissions in the construction sector increased by 108.5 Mt compared to that in 2018. With investment reduction, investment-induced carbon emissions declined by 87.0 Mt in the electronic equipment sector, which was the largest decline in investment-induced emissions, followed by the chemical sector (32.6 Mt). This indicates the shift toward more focused investments in specific sectors to stimulate economic growth.

The demand and carbon emissions of the transport sector changed after the COVID-19 outbreak, with declined expenditure by households but increased expenditure by the government. Self-isolation in response to the pandemic created a novel working pattern that included remote work and meetings. This trend curtailed the transport demand of residents. Household expenditure in transport declined by 11.6% in 2020 because of travel restrictions. In contrast, government consumption in the transport sector was expanded. From 2018 to 2020, the decreased carbon emissions of the transport sector due to the reduced transport demand by households and capital were offset by government consumption, which contributed to an increase of 20 Mt. The increase in government-induced transport carbon emissions was more than 10 times the levels from 2012 to 2017 (1.5 Mt). The abnormally expanded transport demand of the government was because of the tremendous demand for transporting antipandemic and living materials during the lockdown. Due to an overall decreased demand in transport, the total output of the transport sector declined by 0.8% in 2020 compared to the 2018 level.

In general, household expenditure in other sectors has not recovered to the pre-pandemic level, and the contribution of rural and urban household consumption to carbon emission increases was mainly from expenditures in the energy sector. The pandemic in the first quarter of 2020 halted economic activities in China, and lockdown policies in the country greatly depressed household consumption. Household consumption in products of the wholesale, retail, and catering sector and other services sector accounted for 55.2% of total household expenditure in 2020 and was the major expenditure. However, expenditure in the two sectors decreased by 1.4% and increased by only 0.8% during 2018 to 2020. This leads to an overall 4.4% growth in household consumption from 2018 to 2020. Considering that household consumption grew by 5.5% in 2019, this indicates that a shrink in household consumption occurred in 2020 and that consumer expectation has not completely recovered from COVID-19. Along with changes in expenditure, private-induced carbon emissions in many sectors were reduced, such as the food and tobacco, chemical, wholesale, and retail sectors. The increase in carbon emissions in the non-energy sectors was nearly zero. A significant contraction in demand in discretionary purchases, such as clothes and retail, drives the downward trend of carbon reduction by household consumption.

### Rebound in Carbon-Intensive Production and Other Factors

The growth of carbon emissions induced by the production structure toward carbon-intensive production slowed in the new normal, but COVID-19 disrupted this benign trend. In the new normal, the effect of supply side adjustment assisted in the optimization of the production structure, reflected in sectoral emission changes. The elimination of backward production capacity can be seen in the decline of investment-induced emissions in carbon-intensive sectors, such as the electrical equipment, metal products, and ordinary and special equipment sectors. However, the adjusting trend of the production structure toward low-carbon production was disrupted by the pandemic in 2018–2020. In these two years, the production structure contributed to an annual growth rate of 2.9% in the increase of carbon emissions, higher than the average rate (1.3%) in the new normal phase (2012–2018).

The deterioration in the production structure resulted from increased intermediate input intensity and reliance on carbon-intensive input. In 2018 to 2020, intermediate input intensity (the share of intermediate inputs in the total inputs) of several sectors, especially carbon-intensive sectors, was increased, including the petroleum and coking, nonmetallic mineral products, metal products, electricity, construction, and transport sectors. For example, in 2017 and 2018, the intermediate inputs accounted for about 52% of the total inputs, while the proportion was enlarged to 61% in 2020. Consequently, the overall intermediate input intensity of all sectors grew from 56.8% to 57.9% during 2018 to 2020, which was reduced from 2017 to 2018 in contrast. This indicates less value created by the same output, which leads to a reduced production efficiency and usually a lower productivity.^47^ Apart from intermediate input intensity, changes in production structure in 2020 were attributed to the reliance on carbon-intensive inputs, i.e., the increase in the share of carbon-intensive inputs in the total inputs. For example, the intermediate inputs of the petroleum and coking sector and the chemicals sector accounted for 2.2% and 8.2% of the total inputs in all sectors in 2018, respectively, and the proportions were expanded to 2.4% and 8.5% in 2020. To be specific, the transport sector consumed more products from the petroleum and coking sector, increasing from 6.3% to 8.0% during 2018 to 2020, which indicates preference for fossil fuels.

## Discussion

Although the shock of the COVID-19 pandemic halted economic activities in early 2020, the return of economic growth in the latter half of 2020 caused a robust rebound in carbon emissions (Figure 1). We analyzed the changes in the driving forces of carbon emissions in the period 2012–2020 via input–output analyses and SDA. The long-run contribution of energy efficiency, final demand patterns, and industrial updates to China’s carbon emission reductions are also revealed in the literature.^20,48,49^ In summary, structural changes are revealed after the outbreak of the COVID-19, including energy efficiency loss in some heavy industries (metal products, petroleum and other fuels, and nonmetallic mineral products), expansion in export-induced emissions, more concentrated investments in certain sectors, and rebounds in carbon-intensive production.

### The Challenges of Achieving Decarbonization in the Post-Pandemic Era

Efficiency gains have been the predominant force that reduces carbon emissions while carbon intensity reduction was decelerated after the pandemic. The improvements to energy efficiency are mainly due to technological progress as well as energy transformation. The investment in and development of green energy innovation helps cut the cost of cleaner energy. For example, the cost of solar power in China is the lowest in the globe,^50^ at $0.034/kWh in 2021.^51^ Advances in technological evolution facilitate energy efficiency during production as well as transitions in the energy mix. The development of wind and solar power is also the most cost-effective pathway for China to accelerate decarbonization.^52^ Other factors, such as the market revolution shifting from a monopoly market to competition and energy network transmission, also contribute to the improvement of energy efficiency. Nonetheless, the benign trend in the decoupling of China’s economic growth from fossil fuel consumption was impeded by COVID-19 in 2020 because of the drop in energy prices and the reluctance for decarbonization action of companies in light of the urge for economic recovery. The preference for fossil fuels led to risks in the future improvement of energy efficiency. Obstacles in efficiency improvement in the key sectors led to the decelerated decoupling of GDP growth from carbon emissions. Consequently, the carbon intensity reduction of GDP in 2020 was slower than that in the pre-pandemic period. Policy intervention is necessary to adjust the rebounded preference for energy-consumption-supported production and deteriorated energy efficiency. In this regard, motivating energy transitions into renewable energy usage and developing a well-functioning carbon trading mechanism are recommended for future decarbonization.^53^

Carbon emissions driven by economic growth were very much mitigated due to COVID-19, while the rebounded export demand and the shift toward targeted sector-based investments may pave the way for long-term structural impacts of the pandemic on carbon emissions. Since the new normal, carbon emissions induced by capital formation and exports have continued to decline, while household and government consumption have become the main agencies that cause increases in emissions. This trend is accompanied by a shift from heavy industry investment to consumption in services. However, in 2020, the lockdown measurements and travel restrictions reduced household consumption. On the other hand, a shift toward more targeted sector-based investments and expanded exports is revealed after the COVID-19 outbreak. The fiscal stimulus packages led to a more concentrated investment in industries such as infrastructure and construction, and expanded export share boosted some carbon-intensive production. In the post-pandemic era, investments in low-carbon technologies and industries are important to avoid future carbon emission trajectories being locked in the high-carbon industries. Considering that consumption-driven growth induced fewer carbon emissions than investment-induced growth, stimulating private consumption is important for a continuous transition from investment-led growth to consumption-led growth for low-carbon economic development and a green and resilient recovery from COVID-19.

Changes in consumer behavior and work patterns may pave the way for long-term impacts on the final demand pattern. For example, the population’s willingness to consume is negatively impacted by pandemic-induced uncertainty.^54^ This will be difficult to recover before the economic trend is improved and probably leads to an attenuation of consumerist tendencies.^8^ Changes in shopping modes also brings opportunities as well as challenges in carbon emissions reduction. The priority for hygiene concerns increased the demand of chemical products, e.g., plastic packages and sterilizing materials.^8^ In addition, people tend to participate in fewer out-of-home activities, and therefore the boost in online shopping highlights the necessity in reducing the carbon footprints of the logic sector.^55^ Home activities enhanced the influence of social media on consumers, and the contents on social media flatforms again exert greater impacts on consumer choices. This brings opportunities for appropriate inventions to guide consumers’ choices toward sustainable and low-carbon consumption. Online working mode during the pandemic assists to reduce emissions of traveling and occupancy.^56^ Interventions are necessary to take advantage of the positive trends in the changes in consumer behavior and work patterns and prevent the acceleration of trends toward high carbon consumption.

A rebound in carbon-intensive production was revealed after the COVID-19 outbreak. In the new normal phase, China started to chase inclusive and sustainable growth driven by innovation and technology. The previous exclusive pursuit of high-speed growth was abandoned, while stock adjustment and high-quality increases became the goal. In the process of structural upgrades, the elimination of the backward production capacity and supply side reform has been accelerated. However, the search for recovering from the pandemic-associated economic crisis witnessed a rebound in the contribution of production structure to carbon emission growth. This is both a result of higher intermediate input intensity and reliance on carbon-intensive inputs. During 2018 to 2020, more intermediate inputs and especially more carbon-intensive products are required to produce the same number of outputs, indicating lower production efficiency as well as a preference for high-carbon products. The promotion of technology development and energy transitions should be focused on improving production efficiency and increasing the added value of products.

### A Green and Resilient Recovery from the Pandemic

While the determinants of emissions have not been changed, the impacts of COVID-19 can be seen in the rapid growth of carbon-intensive production, the rising contribution of investments and exports to increasing emissions, and slowed-down efficiency gains. Policies need to focus on stimulating the weak consumption of urban and rural households and optimizing the promotion of the low-carbon industry to prevent the deterioration of the production structure.

First, stimulus measures targeting a robust rebound of consumption are urgently needed for the economic recovery from COVID-19. China is eager to prop up economic growth by expanding consumption and domestic demand in the new normal. COVID-19 obstructed progress in increasing private consumption because of lowered income and weakened consumer expectations. The contribution of private consumption to the increase in carbon emissions from 2018 to 2020 was downsized compared with the period from 2012 to 2018. In addition, the carbon emissions from household consumption were primarily induced by energy usage, while transport- and retail-related emissions decreased in 2020, indicating that private consumption in traveling and retail commodities has not recovered from the pandemic. Since the success in containing the first wave of the COVID-19 in early 2020, China has not completely returned to pre-pandemic normality. Consumption-led expansion was still at a low level at the early stage of post-pandemic recovery. Therefore, efforts should be taken to increase the consumption-to-GDP ratio, and improving consumer expectations and boosting domestic consumption toward low-carbon patterns is essential for a resilient recovery from the pandemic.

Second, there is a good opportunity to increase investment in decarbonization technologies and accelerate the development of low-carbon industries to achieve a green and inclusive recovery. To prompt development in key segments, such as artificial intelligence and digital information technology, China has invested in new infrastructure construction. The increased infrastructure investment leads to an increase in carbon emissions caused by capital formation. For emerging economies, increasing infrastructure investment is an appropriate fiscal measure to spur economic recovery. From the perspective of achieving climate targets (carbon peak before 2030 and carbon neutrality before 2060), China should seize the opportunity and increase its investment in green technologies and industries to gain competitiveness in decarbonization in the future, for example, supporting the low-carbon transition and promoting the green and sustainable finance of private companies. This would also produce jobs in low-carbon industries and help to prepare for the demand for skilled labor in related industries. There might be potential conflicts considering it is also suggested to change the investment-led economic growth into consumption-led growth, but they are not necessarily at odds. Investment in green industries here requires the shift of current investment patterns toward boosting the development of low-carbon technologies and industries, rather than simply increasing the total amount of investments. Consumption-led growth requires interventions to stimulate private and public consumption instead of suppressing investment.

Third, encouraging innovation and improving the proportion of high-value-added products in exports are crucial to enhancing the position of China’s manufacturing in the global supply chain. With the rising production cost in China due to the shortage of cheap labor and restrictions on carbon reduction, the risk of industrial relocation has been mounting. The development of sophisticated manufacturing is the key to expanding China’s presence in the global market in the future. In 2020, the carbon emissions of exports were heightened compared with the 2018 level for the first time since the new normal. With the booming demand as the rest of the world was still suffering from the pandemic in the second half of 2020, the prosperity of exports in 2020 provided a good chance to enhance the comparative competitiveness of China’s manufacturing. Policies should target high-value-added and low-carbon industries and improve competitiveness in the global market to prevent the rebounding of carbon-intensive and unsustainable production.

## Supporting Information Available

Additional figures, tables, and discussions regarding methods, uncertainty analyses, and the results of pre-COVID emissions driving factors (PDF)
