# Supplementary material for: Factors Driving China’s Carbon Emissions after the COVID-19 Outbreak
Source: Environ Sci Technol. 2023 Nov 16;57(48):19125–36. doi: 10.1021/acs.est.3c03802 (PMC10702430; doi:10.1021/acs.est.3c03802)
Supplement: Supplementary file 1 — es3c03802_si_001.pdf [file es3c03802_si_001.pdf]

Supporting Information for:

Factors driving China's carbon emissions after the COVID-19 outbreak

Xinlu Sun [1], Zhifu Mi[1,*]

[1]The Bartlett School of Sustainable Construction, University College London, London WC1E 7HB, UK

[*]Corresponding author: z.mi@ucl.ac.uk (Z Mi)

In this file:
Number of pages (including this cover page): 19
Number of figures: 8
Number of tables: 2

Supporting Discussion

Uncertainty analysis

As stated in the context, we discussed uncertainties cause by limitation in data and the SDA method. Here, we conduct several analyses to quantify the uncertainty, especially the uncertainty cause by the SDA method. First, regarding the uncertainty cause by sectoral aggregation, we further aggregate the 20 sectors in the context into seven sectors, including the agriculture, mining, industry, energy, construction, transport and services sectors. The result shows that the changes in driving factors of carbon emissions happened after the COVID-19 in consistence with the changes indicated in the context (Fig S1). Secondly, we use the average value of the 5! = 120 equivalent decomposition forms in the context, and here for uncertainty analysis, we calculate all values of the 120 forms to show the disparities. The contribution of the five factors for the emissions growth in 2017-2018 and 2018-2020 is showed, by setting 2017 and 2018 as the benchmark year in the two periods. The results confirm the robustness of the changes in each driving factor as in the context (Fig S2).

Slowdown of China's carbon emissions increases

The growth rate of China's carbon emissions did not follow a constant trend (Fig. S5A). Overall, the path of the increase can be divided into four phases during this period. Before the global financial crisis, China's carbon emissions experienced a high-speed rise because of growing economic development and carbon-intensive exports. The average increase rate of production-based carbon emissions was 17.8% annually from 2002 to 2007. This made China the largest carbon emitter in the world in 2006 [35–37]. The shock of the global financial crisis in 2008 greatly reduced global demand and slowed the increase in carbon emissions in China (3.4%). Entering the postcrisis era, the Chinese government released a series of stimulus packages to boost a robust economic recovery. A four trillion-yuan stimulus plan targeting some carbon-intensive sectors, including infrastructure and construction, was announced to bolster economic expansion. The economic stimulus strategy not only helped the country escape the quagmire of the economic crisis but also led to an intense rebound of carbon emissions growth. From 2008 to 2011, the average growth rate of China's carbon emissions was 9.7% annually. A tipping point of economic development appeared after a rapid recovery from the financial crisis as China entered the "new normal" in 2012-2013, which meant lower economic growth rate but higher quality. With a retarded GDP growth rate, production-based carbon emissions peaked in 2013 at 9.8 Gt and then continued to decline in 2014 and 2015. Carbon emissions were reduced by 3% in this period. The reduction in carbon emissions in this period attracted much attention from academia as it confirmed the feasibility of achieving a low-carbon transition while

maintaining relatively high GDP growth in China. However, the carbon peak in 2013 was a temporary accomplishment, and carbon emissions rebounded after 2017.

Overall, the rapid growth of carbon emissions has ended since the beginning of the new normal. Before 2012, carbon emissions increased by 17% per year, while after 2012, the annual increase rate was drastically reduced to 1.5%. The stabilized carbon emissions were attributed to the decoupling of economic development from carbon-intensive production more than to slowed GDP growth and therefore reflected the characteristics of the new normal phase, with lower speed but higher quality of economic growth and strong policies on carbon reduction. Changes in carbon intensity, which is carbon emissions per unit of GDP, indicate that the carbon reduction from 2013 to 2016 was mainly due to the dramatic decline in carbon intensity. In terms of the sources of carbon emissions, curtailed coal usage was the effective pathway for decarbonization in this period (Fig. S5B). Consequently, the carbon intensity was substantially reduced by 21% during 2013-2016. In contrast, carbon intensity remained nearly constant in the post-financial-crisis era. From 2008 to 2011, carbon intensity was curtailed by only 3%. The difficulty in decarbonization in this period was because of the urgency of achieving economic recovery and extensive investment in energy-intensive sectors. In recent years, China has encountered a bottleneck period in carbon abatement as marginal abatement increases. From 2017, when carbon emissions started to rebound, the carbon intensity was reduced by 8%, which was much slower than the earlier stage of the new normal phase.

Direct carbon emissions from household energy consumption have also plateaued in recent years. Household carbon emissions increased from 192 Mt to 448 Mt from 2002 to 2017 because of the increasing energy demand (Fig. S5C). The rising energy consumption of urban households was the main reason for increased carbon emissions. The purchasing power and carbon-intensive lifestyle of urban households as well as rapid urbanization resulted in the contribution of urban households to direct carbon emissions. A clear transition of the energy mix is revealed, and carbon emissions induced by the coal consumption of both urban and rural households have been critically reduced. Urban households have successfully switched from coal usage to gas for their essential life demands. In 2002, carbon emissions from urban coal and gas usage were 51 Mt and 10 Mt, respectively. The roles of coal and gas have been reversed since 2010, and carbon emissions from urban coal and gas usage were 12 Mt and 120 Mt in 2020, respectively. Access to gas in rural areas in China has been a problem that obscures rural energy transitions because of rural households' scattered inhabitation and distance from the gas grid. However, rural coal-induced carbon emissions peaked in 2015 at 122 Mt and continued to decrease to 85 Mt in 2020. The reduction in coal usage was mainly due to the electrification of rural household energy consumption. From 2002 to 2020, rural electricity consumption increased from 67 billion kWh to 524 billion kWh. Nonetheless, coal usage is still the main resource for rural carbon emissions, and therefore, the accessibility of clean and high-quality energy is still a challenge in rural China. Oil-induced carbon emissions have been increasing in both urban and rural areas, which is mainly attributed to gasoline and liquefied petroleum gas

(LPG) usage. Urban and rural residents use LPG for cooking when natural gas is difficult to access. The increase in LPG usage has been stabilized because of progress in gas pipeline construction. Gasoline continued to increase drastically with the rapid expansion of private car ownership. Reducing the carbon emissions induced by household oil consumption requires policies that target the transition of oil fuel vehicles towards new energy vehicles as well as encouraging more responsible consumption behaviours with regard to low-carbon transport.

Determinants of the carbon emissions change before COVID-19

Apart from the three stages in the main context, we further divide the ten years from 2002 to 2012 into two earlier stages by a five-year window. The first stage is the rapid increase stage after accession to the WTO (2002-2007) and the second stage is the post-financial-crisis era, when carbon emissions rebounded (2007-2012).

In the long run, the improvement of energy efficiency has been the sole factor that drives the decarbonization of China's economy (Fig. S6). From 2002 to 2020, the contribution of energy efficiency to emission reduction was 188%, which means that carbon emissions per unit of total output have been significantly decreased. The continuously declining carbon intensity is mainly achieved by progress in low-carbon technology energy and the elimination of backward production capacity. From 2002 to 2012, efficiency gains in the manufacturing sector and some light industries, including equipment production sectors, food, textiles, and paper, contributed to 99% of the carbon reduction in China (Fig. S7A-D). After the financial crisis, the advantage of energy efficiency was slightly weakened. One of the main reasons was the deterioration in carbon reduction of the energy sector, including electricity, gas and water production and supply. From 2007 to 2012, the carbon intensity of the energy sector rose by 3%. Due to the supply-side adjustment and the elimination of backward production capacity, energy efficiency has been enhanced in the new normal (from 33% during 2007-2012 to 49% during 2012-2017). In this period, the carbon intensity of most sectors decreased immensely. For instance, the carbon intensity of the "Petroleum, Coking, Nuclear Fuel" sector declined by 49% in the new normal, while this figure was only 17% in 2007-2012, indicating that the energy efficiency improvement almost tripled. In 2017-2018, energy efficiency improvements accelerated, with efficiency gains in some sectors, including the construction sector, transport sector, chemical sector, and energy sector. Although it has been the major driving force of decarbonization in China for decades, the potential for energy efficiency improvements has been constricted with the transformation of the energy mix and technology updates. The annual contribution of efficiency gains to carbon reduction was as high as 13.2% in 2002-2007 but drastically declined to 3.5% in the following stage from 2007 to 2012. The loss of efficiency advantage gradually recovered in the new normal phase to an annual contribution rate of 3.7% due to the decisive supply-side reform.

After driving up the increase in carbon emissions for ten years from 2002-2012, consumption patterns started to become a decarbonization force during 2012-2017 and then recently reversed again. The contribution of consumption patterns is in accordance with the consumption structure caused by different final users, namely, rural and urban households, government, capital and inventory, and exports. In general, a clear shift of the driving forces of carbon emissions from capital formation to household consumption is revealed (Fig. S5D).

Before the new normal, the accelerated economic growth as well as the tremendous investment for the recovery from the financial crisis drove the growth of capital formation and induced carbon emission increases. From 2002 to 2012, carbon emissions caused by the final demand of capital and inventory changes increased by 4358 Mt, accounting for 77% of the total carbon increase. The reliance on international trade and expanded export demand, especially before the financial crisis, led to an increase of 1075 Mt carbon emissions from 2002 to 2007. With the search for an inclusive and sustainable industry structure, China strengthened its efforts to prevent the disorderly expansion of capital and promoted supply-side transformation to optimize the industry structure in the new normal. Consequently, carbon emissions induced by capital formation largely declined. Carbon emissions induced by exports also decreased in the new normal stage because of rising labour costs and restricted sustainability requirements. The contribution of private and government consumption has been enhanced since then. However, the decarbonization effect by consumption patterns was reversed again since 2017 with the consumption caused by the rebounded contribution of capital formation. The incremental carbon emissions induced by capital and changes in inventory increased from 42% in 2012-2017 to 59% in 2017-2018. This trend continued in 2020 as consumer confidence had not completely recovered.

Per capita consumption volume, production structure, and population have driven the growth of carbon emissions in the whole period under consideration. Consumption volume, indicating the changes in GDP growth, is the predominant factor that accounts for the rise in carbon emissions. With the entrance of the new normal, the pursuit of lower speed but higher-quality economic development also slowed emission expansion. The production structure contributed to the increase in China's carbon emissions, but the contribution was constricted in the new normal. From 2002 to 2007, the production structure explained a 78% increase in carbon emissions, and the contribution of the production structure to carbon emissions was condensed to 29% during 2007-2012. The increase in carbon emissions of the construction sector by capital formation was the main cause of the increase in China's carbon emissions (Fig. S8). Being policy-sensitive and capital-driven, the expansion of the construction sector before 2012 was mainly due to the rapid development of the real estate market as well as the economic stimulus package targeting high-speed rail network and infrastructure construction after the financial crisis. This also led to the expansion of related sectors, for example, the transport equipment sector that produces high-speed trains. The accession to the WTO boosted the manufacturing sectors in China because of the large demand for exports. Consequently, carbon emissions

induced by exports of ordinary and special equipment, transport equipment and chemicals increased considerably in this period. The extensive carbon emissions of the construction sector and several manufacturing sectors, driven by capital formation and exports, explained most of the total increase in this period. In the new normal phase, the contribution of the production structure to the carbon emission increase was less and therefore was offset by rapid energy efficiency improvement. The growing trend of the population is rather stable and contributes to a growth rate of emissions of 1.2% annually.

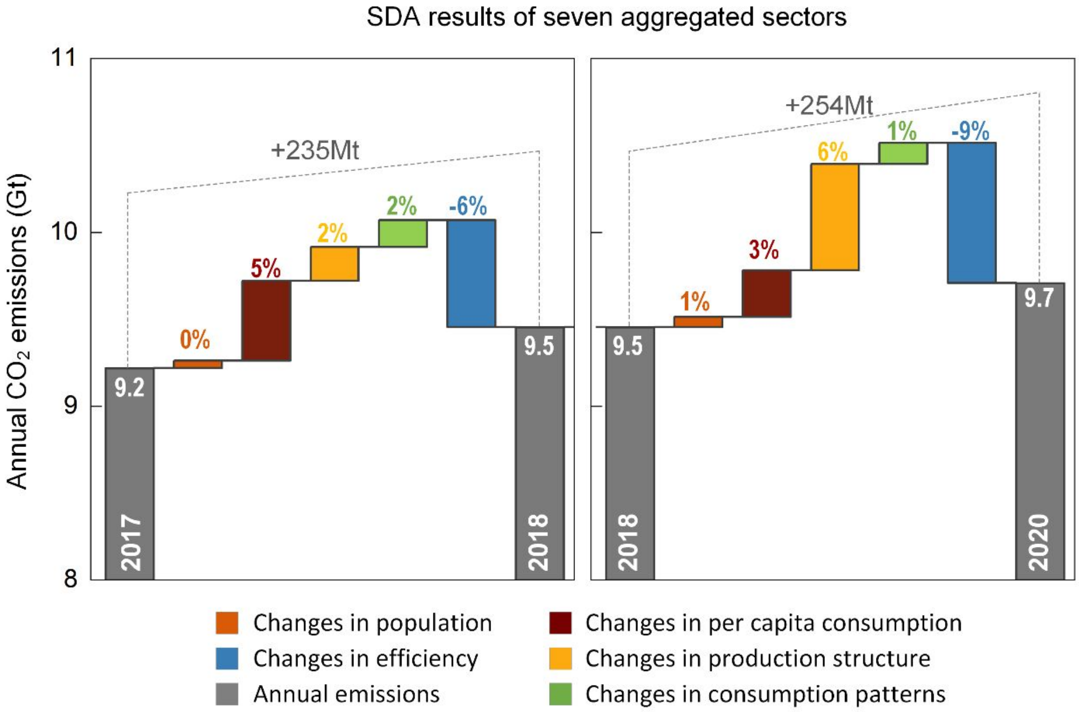

Fig. S1. Absolute contributions of different factors to changes in Chinese CO_2 emissions for 2017–2018 and 2018–2020. The 20 sectors are further aggregated into seven sectors.

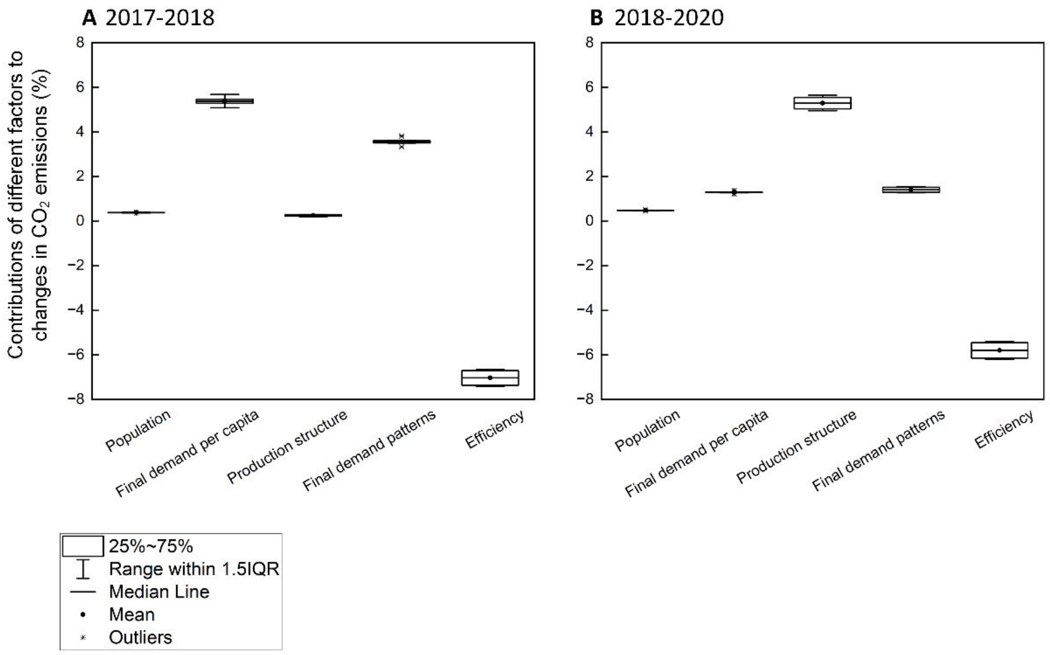

Fig. S2. Descriptive statistics for the contributions of five factors to energy intensity changes across 120 alternative decomposition forms between two time periods. A. 2017-2018. B. 2018-2020.

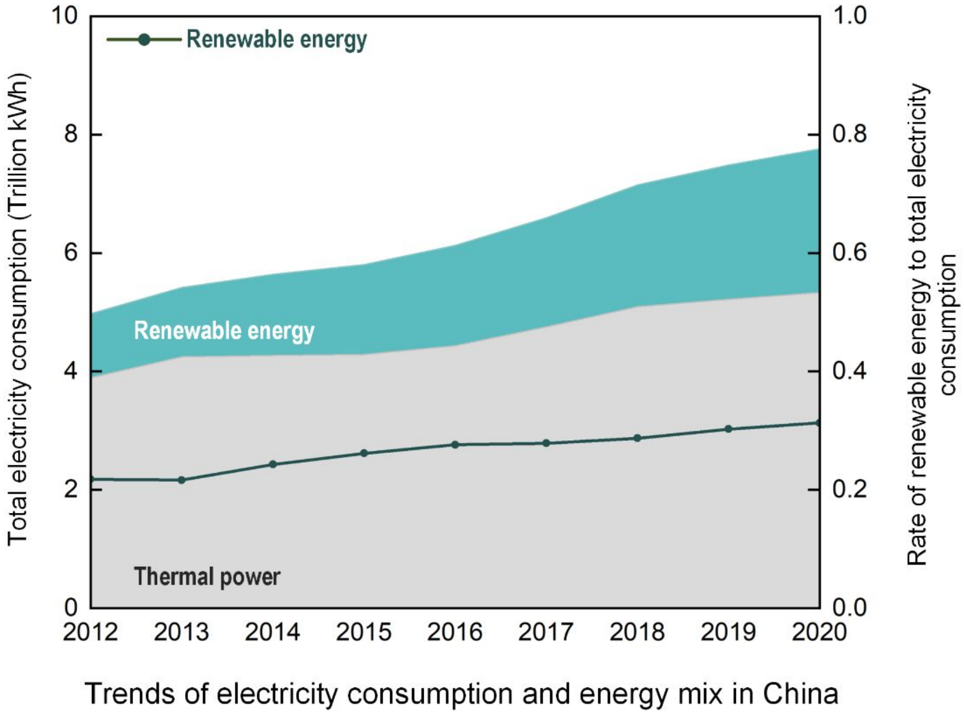

Fig. S3. Trends of electricity consumption and energy mix in China 2012-2020. The line shows rate of renewable energy to total electricity consumption, and areas show the volume of electricity consumption.

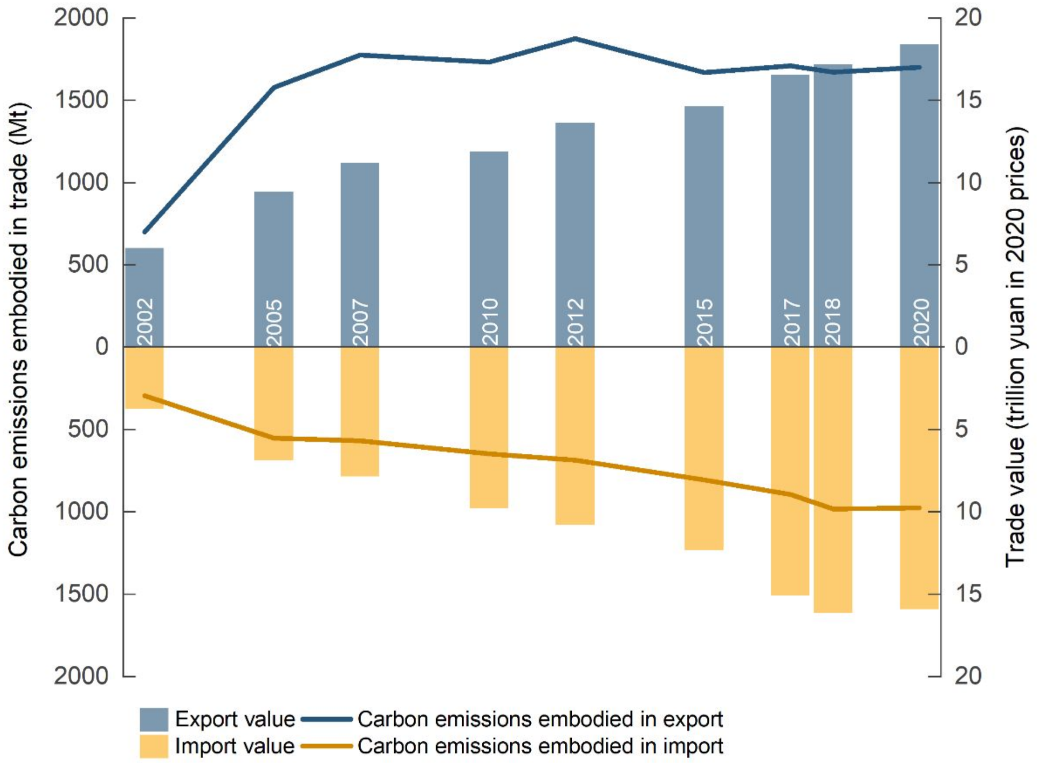

Fig. S4. Value of export and import and the embodied carbon emissions in international trade in China 2012-2020.

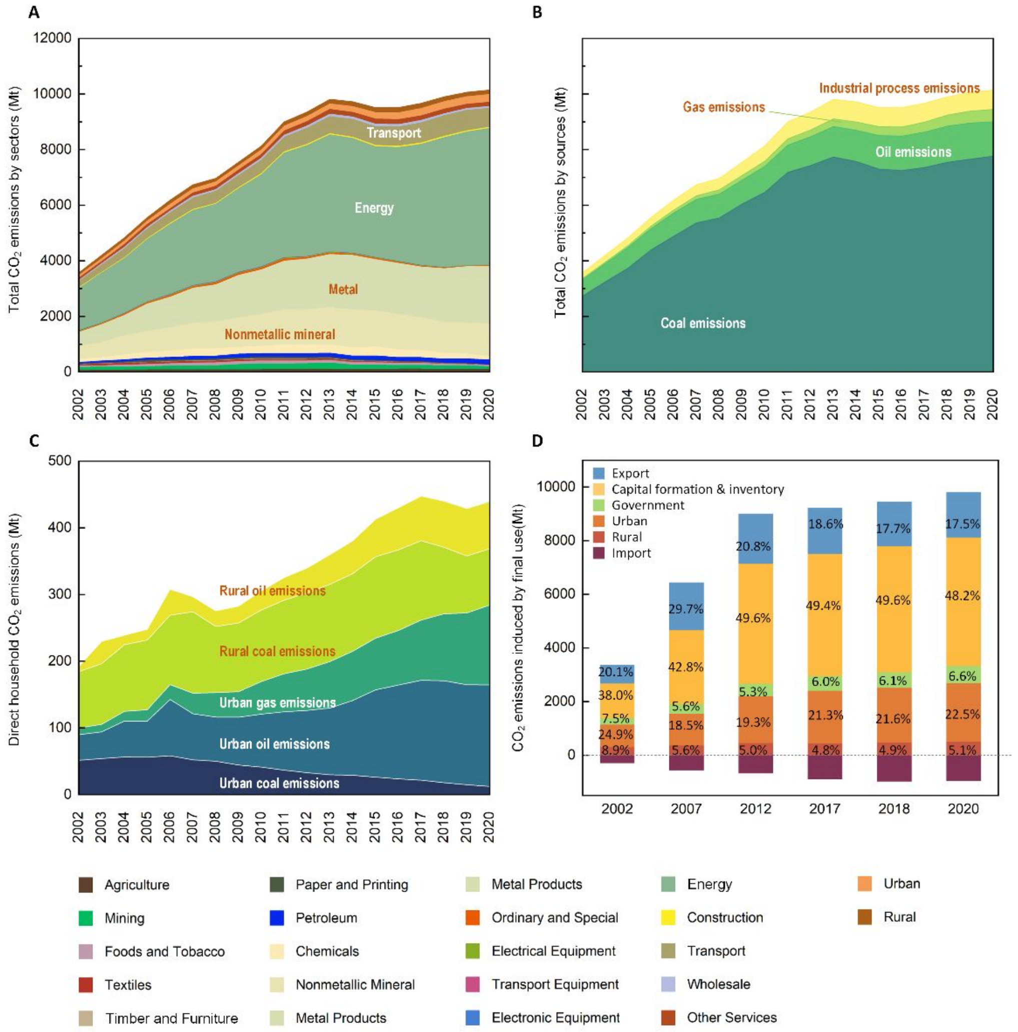

Fig. S5. Trends of China's carbon emissions from 2002-2020. A. Trends of carbon emissions by sectors. B. Trends of carbon emissions by fuel. C. Direct household CO_2 emissions in China. CO_2 emissions induced by different final uses (rural consumption, urban consumption, government consumption, capital formation, inventory changes and exports).

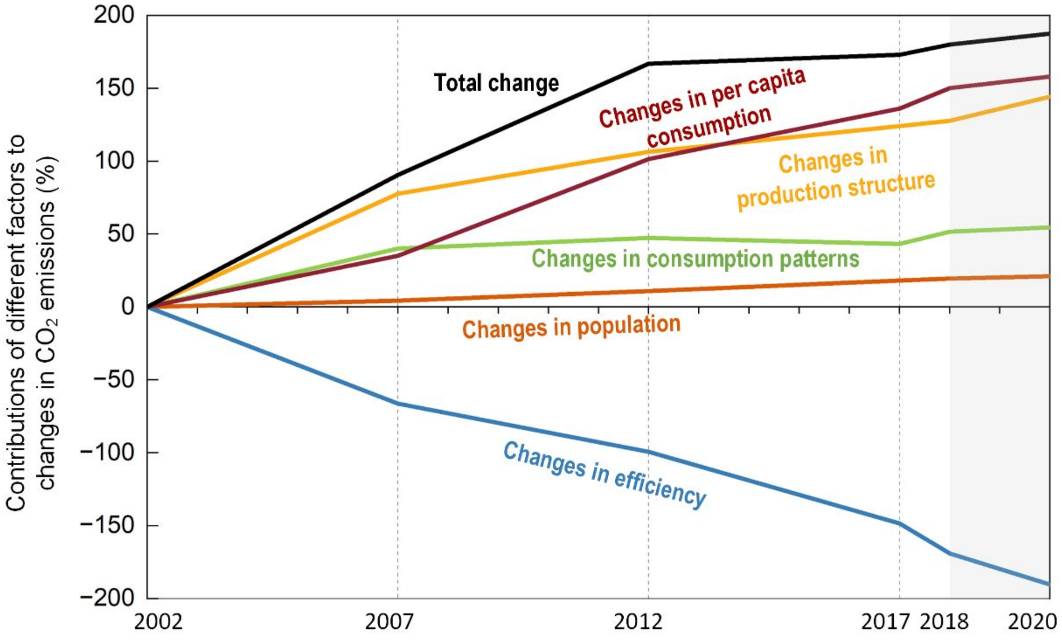

Fig. S6. Relative contributions of different factors to changes in Chinese CO2 emissions for all stages. A. 2002-2007. B. 2007-2012 C. 2012-2017. D. 2017-2018 and 2018-2020.

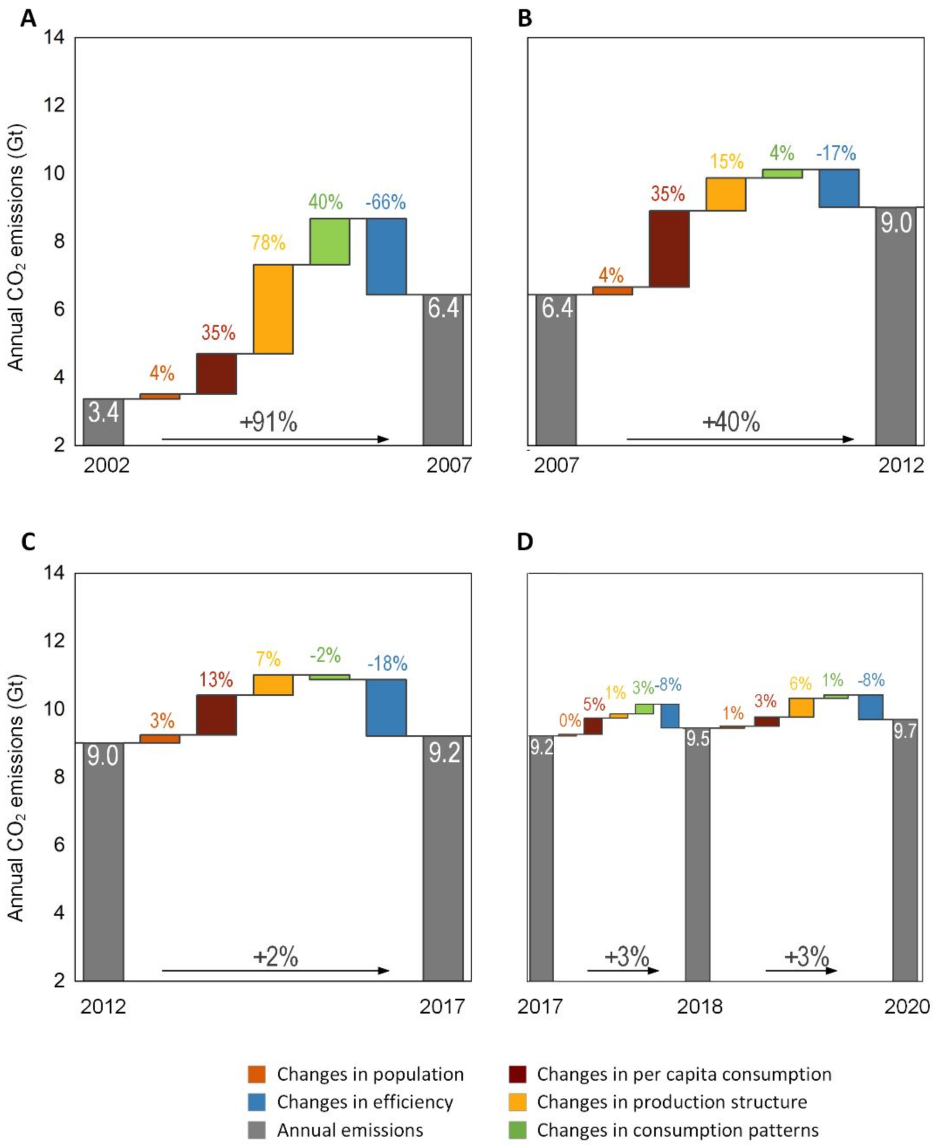

Fig. S7. Absolute contributions of different factors to changes in Chinese CO2 emissions for all stages. A. 2002-2007. B. 2007-2012 C. 2012-2017. D. 2017-2018 and 2018-2020.

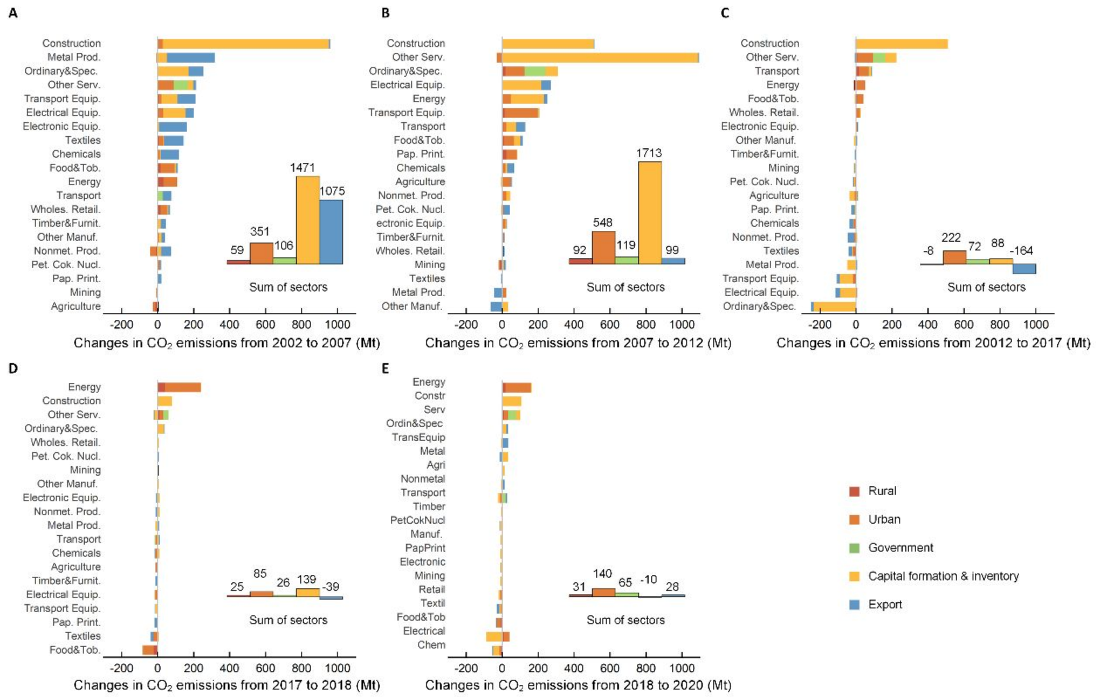

Fig. S8. Contributions of different sectors and final uses to Chinese CO2 emissions growth from 2002 to 2020. A, B, C, D and E show the results for 2002–2007, 2007–2012, 2012–2017, 2017-2018, and 2018-2020, respectively.

Table S1 CO_2 emission factors for energy consumption

No.	Energy types	Emission factors (Mt CO_2 / 10^4 t, 10^8 m^3)
1	Raw coal	0.0162
2	Cleaned coal	0.0204
3	Other washed coal	0.0119
4	Briquettes	0.0138
5	Coke	0.0288
6	Coke oven gas	0.1153
7	Other gas	0.0596
8	Other coking products	0.0252
9	Crude oil	0.03
10	Gasoline	0.0293
11	Kerosene	0.0304
12	Diesel oil	0.0309
13	Fuel oil	0.0317
14	Liquefied petroleum gas (LPG)	0.0313
15	Refinery gas	0.0334
16	Other petroleum products	0.0303
17	Nature gas	0.2161
18	Non-fossil Heat	0
19	Non-fossil Electricity	0
20	Other energy	0

Table S2 Concordance of sectors for Chinese IO tables, carbon emission inventories and Exiobase

MRIO tables.	Carbon emission inventories	Exiobase MRIO tables
Agriculture	Farming, Forestry, Animal Husbandry, Fishery and Water Conservancy	Cultivation of paddy rice, wheat, cereal grains n.e.c, vegetables, fruit, nuts, oil seeds, sugar cane, sugar beet, plant-based fibers, crops n.e.c;<br>Cattle, pigs, poultry farming, meat animals n.e.c, animal products n.e.c, raw milk;<br>Wool, silk-worm cocoons; Manure treatment (conventional), storage and land application; Manure treatment (biogas), storage and land application; Forestry, logging and related service activities; Fishing, operating of fish hatcheries and fish farms; service activities incidental to fishing
Mining	Coal Mining and Dressing; Petroleum and Natural Gas Extraction; Ferrous Metals Mining and Dressing; Nonferrous Metals Mining and Dressing; Nonmetal Minerals Mining and Dressing; Other Minerals Mining and Dressing	Mining of coal and lignite; extraction of peat; Extraction of crude petroleum, natural gas, and services related; Extraction, liquefaction, and regasification of other petroleum and gaseous materials<br>Mining of uranium and thorium ores, iron ores, copper ores, nickel ores, aluminium ores, precious metal ores, lead, zinc and tin ores, other non-ferrous metal ores, and concentrates;<br>Quarrying of stone, sand and clay;<br>Mining of chemical and fertilizer minerals, production of salt, other mining and quarrying n.e.c
Foods and Tobacco	Food Processing<br>Food Production<br>Beverage Production<br>Tobacco Processing	Processing of meat cattle, meat pigs, meat poultry, meat products n.e.c, vegetable oils and fats, dairy products, food products n.e.c; Processed rice; Sugar refining; Manufacture of beverages, fish products, tobacco products
Textiles	Textile Industry; Garments and Other Fiber Products; Leather, Furs, Down and Related Products	Manufacture of textiles, wearing apparel; Dressing and dyeing of fur;<br>Tanning and dressing of leather; Manufacture of luggage, handbags, saddlery, harness and footwear
Timbers and Furniture	Logging and Transport of Wood and Bamboo; Timber Processing, Bamboo, Cane, Palm Fiber & Straw Products;	Manufacture of wood and of products of wood and cork, except furniture; Manufacture of articles of straw and plaiting materials; Re-processing of secondary wood

	Furniture Manufacturing	material into new wood material
Paper and Printing	Papermaking and Paper Products; Printing and Record Medium Reproduction; Cultural, Educational and Sports Articles	Pulp; Re-processing of secondary paper into new pulp; Paper; Publishing, printing and reproduction of recorded media
Petroleum, Coking, Nuclear Fuel	Petroleum Processing and Coking	Manufacture of coke oven products; Petroleum Refinery; Processing of nuclear fuel
Chemicals	Raw Chemical Materials and Chemical Products; Medical and Pharmaceutical Products; Chemical Fiber; Rubber Products; Plastic Products	Plastics, basic; Re-processing of secondary plastic into new plastic; N-fertiliser; P- and other fertilizer; Chemicals n.e.c; Manufacture of rubber and plastic products
Nonmetallic Mineral Products	Nonmetal Mineral Products	Manufacture of glass and glass products; Re-processing of secondary glass into new glass; Manufacture of ceramic goods, bricks, tiles and construction products, in baked clay, cement, lime and plaster; Re-processing of ash into clinker; Manufacture of other non-metallic mineral products n.e.c.
Metal Products	Smelting and Pressing of Ferrous Metals<br>Smelting and Pressing of Nonferrous Metals<br>Metal Products	Manufacture of basic iron and steel, ferro-alloys, precious metals, aluminum, lead, zinc and tin, copper, and other non-ferrous metal; Re-processing of secondary metal into new; Casting of metals; Manufacture of fabricated metal products, except machinery and equipment.
Ordinary and Special Machinery	Ordinary Machinery<br>Equipment for Special Purposes	Manufacture of machinery and equipment n.e.c.
Transport Equipment	Transportation Equipment	Manufacture of motor vehicles, trailers and semi-trailers, and other transport equipment
Electrical Equipment	Electric Equipment and Machinery	Manufacture of electrical machinery and apparatus n.e.c.
Electronic Equipment	Electronic and Telecommunications Equipment	Manufacture of office machinery and computers<br>Manufacture of radio, television and communication equipment and apparatus

Other Manufacturing Industry	Instruments, Meters, Cultural and Office; Machinery; Other Manufacturing Industry; Scrap and waste	Manufacture of medical, precision and optical instruments, watches and clocks; Manufacture of furniture; manufacturing n.e.c; Recycling of waste and scrap, and bottles by direct reuse.
Electricity, Gas, Water	Production and Supply of Electric Power, Steam and Hot Water; Production and Supply of Gas and Tap Water	Production of electricity by coal, gas, nuclear, hydro, wind, petroleum and other oil derivatives, biomass and waste, solar photovoltaic, solar thermal, tide, wave, ocean, Geothermal, n.e.c; Transmission, distribution and trade of electricity; Manufacture and distribution of gas; Steam and hot water supply; Collection, purification and distribution of water
Construction	Construction	Construction; Re-processing of secondary construction material into aggregates
Transport	Transportation, Storage, Post and Telecommunication Services	Sale, maintenance, repair of motor vehicles, motor vehicles parts, motorcycles, motorcycles parts and accessoiries; Retail sale of automotive fuel; Transport via railways; Other land transport; Transport via pipelines; Sea and coastal water transport; Inland water transport; Air transport; Supporting and auxiliary transport activities; activities of travel agencies; Post and telecommunications
Wholesale, Retail, Catering	Wholesale, Retail Trade and Catering Services	Wholesale trade and commission trade, except of motor vehicles and motorcycles; Retail trade, except of motor vehicles and motorcycles; repair of personal and household goods; Hotels and restaurants
Other Services	Others	Financial intermediation, insurance and pension funding, activities auxiliary to financial intermediation; Real estate activities; Renting; Computer and related activities; Research and development; Other business activities; Public administration and defence; compulsory social security; Education; Health and social work; Incineration of waste; Biogasification of waste; Composting of food waste, paper and wood, incl. land application; Waste water treatment; Landfill of waste; Activities of membership organisation n.e.c; Recreational, cultural and sporting activities; Other service activities; Private households with employed persons; Extra-territorial organizations and bodies
